# Supplementary material for: Non-canonical NOTCH3 signalling limits tumour angiogenesis
Source: Nat Commun. 2017 Jul 18;8:16074. doi: 10.1038/ncomms16074 (PMC5520050; doi:10.1038/ncomms16074)
Supplement: Supplementary Information [file ncomms16074-s1.pdf]

File Name: Supplementary Information
Description: Supplementary Figures and Supplementary Table

File Name: Peer Review File
Description:

Supplementary Table 1

Primers and taqman probes used were the following:

mHPRT	5'tcctcctcagaccgcttt	5'cctggttcatcatcgctaatc	95
mCD 31	5'gctggtgctctatgcaagc	5'atggatgctgttgatggtga	64
mDll-4	5'aggtgccacttcggttacac	5'gggagagcaaatggctgat	106
mSMA	5'ctctcttccagccatctttcat	5'tataggtggtttcgtggatgc	58
mVEGFR 2	5'cagtggtactggcagctagaa	5'acaagcatacgggcttgttt	68
mVEGF A	5'ttaaacgaacgtacttgcaga	5'agaggtctggttcccgaaa	4
hHPRT	5'tgacactggcaaaacaatgca	5'gctcctttcaccagcaagct	73
hJagged-1	5'caggacctggttaacggattt	5'gcctcacatttgcatc	48
hNotch3	5'gccaagcggctaaaggta	5'cactgacggcaatccaca	30
hCD31	5'gcaacacagtccagatagtcgt	5'gacctcaaactgggcatcat	14

a

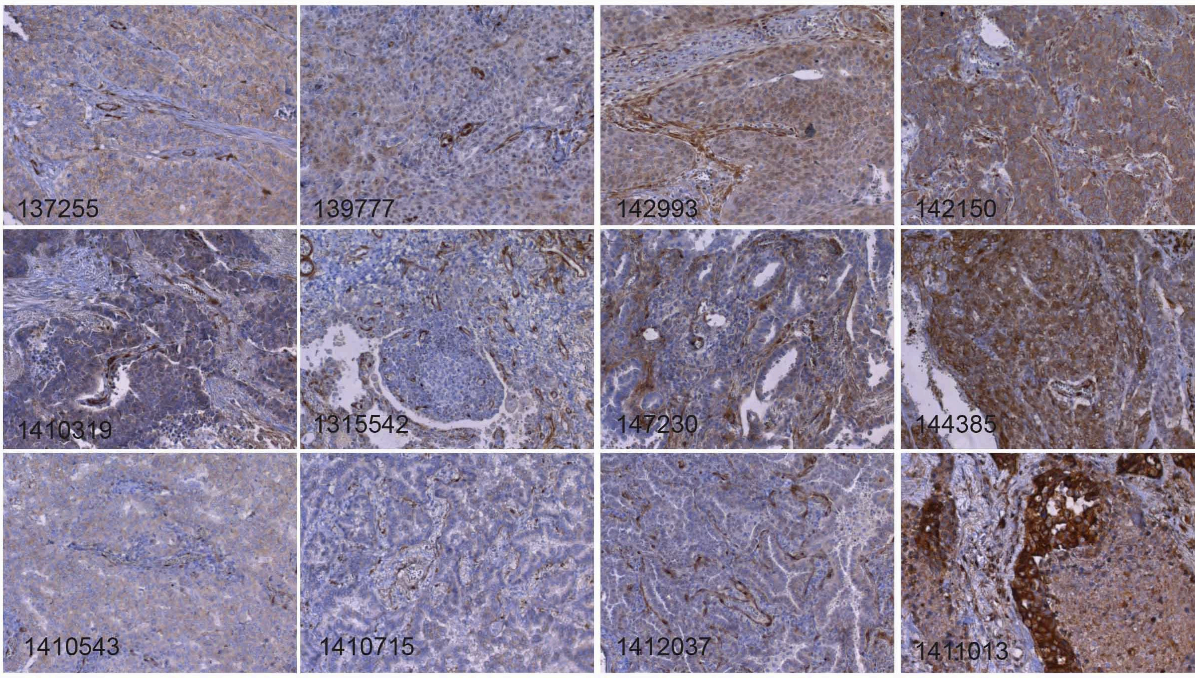

b

Histology	Tumor tissue						Vasculature
	Nucleus		Cytoplasm		Membrane		
	%	Intensity	%	Intensity	%	Intensity	
SCC	0	0	50	++	40	++	+++
SCC	50	0	70	+	70	+	+++
SCC	0	0	90	++	30	++	+++
SCC	0	0	60	++	10	++	+++
SCC	80	+	20	+	10	++	+++
SCC	0	0	90	++	0	0	+++
SCC	70	+	50	++	5	++	+++
SCC	80	++	90	++	40	++	+++
SCC	70	++	100	++	10	+	+++
SCC	10	+	40	++	60	+	+++
ADC	0	0	0	0	0	0	++
ADC	0	0	40	+	0	0	++
ADC	0	0	50	+	0	0	+++
ADC	0	0	70	+++	30	+	+++
ADC	60	+	0	0	0	0	+++
ADC	30	+	30	+	0	0	+++
ADC	10	+	30	+	20	+	+++
ADC	0	0	0	0	60	++	+++
ADC	0	0	10	+	10	+	+++
ADC	10	+	5	+	5	+	+++
ADC	10	+	40	+	0	0	+++

Supplementary Figure 1: Notch3 expression in NSCLC patients. a, Representative images from Notch3 immunohistochemistry performed on tumour section from NSCLC lung cancers patients showing the diversity of Notch3 expression in the tumour compartment and the constant staining of Notch3 in the tumour vasculature. **b**, Quantification of the expression of Notch3 in 10 squamous cell carcinomas and 11 adenocarcinomas from NSCLC patients.

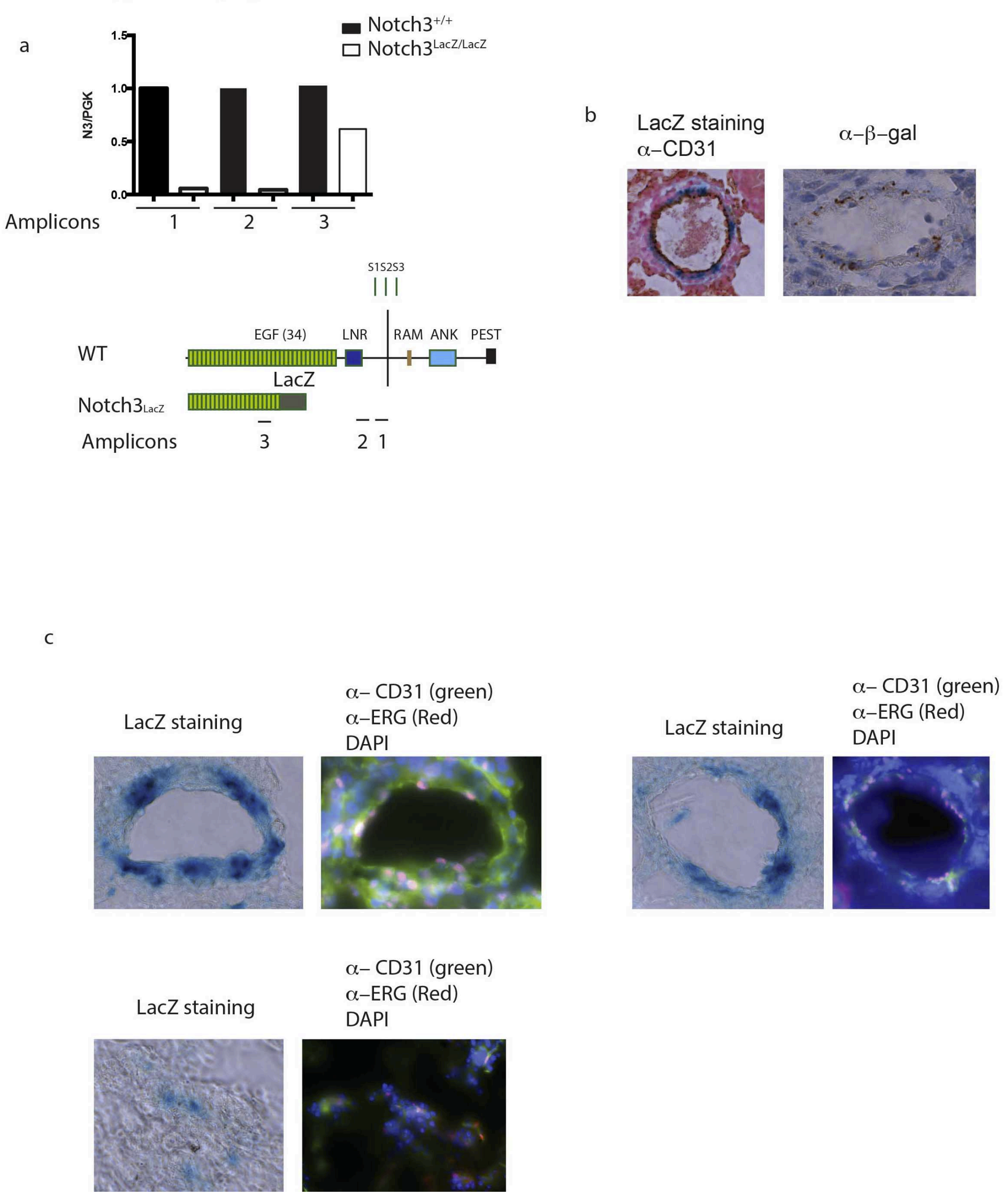

Supplementary Figure 2: Notch3 expression in the Notch3$^{LacZ/+}$ mice. a, Notch3 mRNA expression determined with 3 different primer pairs amplifying three amplicons on the Notch3 mRNA in wild type mice or Notch3$^{lacZ/LacZ}$ mice. **b**, Comparison of the LacZ staining with the immunohistochemistry staining with anti-β-galactosidase antibody showing the specificity of the β-galactosidase enzymatic reaction. **c,** Raw images from LacZ staining and immunofluorescence corresponding to Fig. 1c.

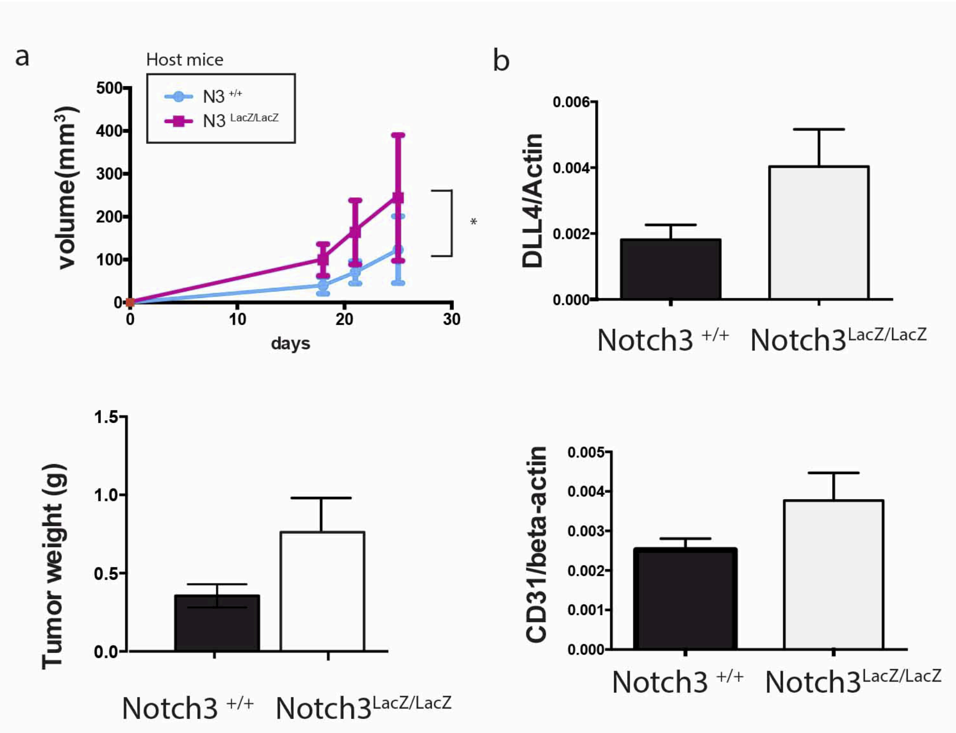

Supplementary Figure 3: Notch3 limits tumour growth and vascularization *in vivo*.

a, 5.10^5 EO771 cells were implanted into the left flank of wild-type C57Bl/6 mice ($N3^{+/+}$, n=4) or of Notch3 LacZ homozygous Knock-in C57Bl/6 littermates ($N3^{LacZ/LacZ}$, n=4). Tumour growth was monitored from day 16 until day 24 when mice were sacrificed. Two-Way ANOVA was performed to assess Time and Genotype effect on tumour growth (Time: p<0,0001; Genotype: p=0,0028). **b**, mRNA was extracted from tumours dissected after 25 days of growth from wild-type C57Bl/6 mice ($N3^{+/+}$, n=4) or Notch3 mutant mice $N3^{LacZ/LacZ}$ C57Bl/6 littermates ($N3^{LacZ/LacZ}$, n=4). Quantitative RT-PCR was performed to measure CD31, DLL4, expression (means +/- SD).

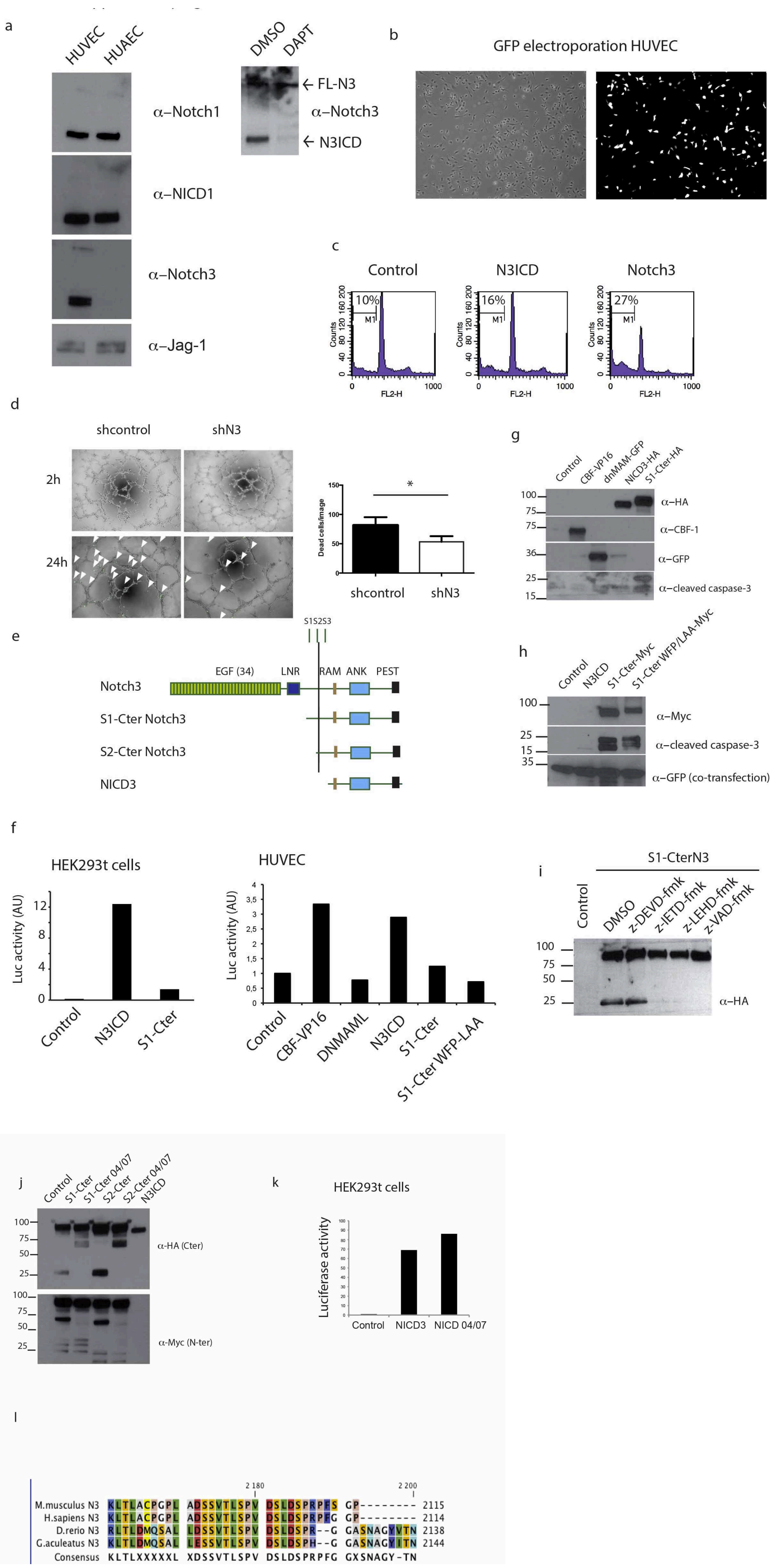

a
HUVEC
HUAEC
α–Notch1
α–NICD1
α–Notch3
α–Jag-1
DMSO
DAPT
FL-N3
α–Notch3
N3ICD
b
GFP electroporation HUVEC
c
Control
N3ICD
Notch3
10%
16%
27%
M1
Counts
FL2-H
d
shcontrol
shN3
2h
24h
Dead cells/image
shcontrol
shN3
e
S1S2S3
EGF (34)
LNR
RAM
ANK
PEST
Notch3
S1-Cter Notch3
S2-Cter Notch3
NICD3
f
HEK293t cells
Luc activity (AU)
Control
N3ICD
S1-Cter
HUVEC
Luc activity (AU)
Control
CBF-VP16
DNMAML
N3ICD
S1-Cter
S1-Cter WFP-LAA
g
Control
CBF-VP16
dnMAM-GFP
NICD3-HA
S1-Cter-HA
α–HA
α–CBF-1
α–GFP
α–cleaved caspase-3
h
Control
N3ICD
S1-Cter-Myc
S1-Cter WFP/LAA-Myc
α–Myc
α–cleaved caspase-3
α–GFP (co-transfection)
i
S1-CterN3
Control
DMSO
z-DEVD-fmk
z-IETD-fmk
z-LEHD-fmk
z-VAD-fmk
α–HA
j
Control
S1-Cter
S1-Cter 04/07
S2-Cter
S2-Cter 04/07
N3ICD
α-HA (Cter)
α-Myc (N-ter)
k
HEK293t cells
Luciferase activity
Control
NICD3
NICD 04/07
l
M.musculus N3 KLTLACPGPL ADSSVTLSPV DSLDSPRPFS GP-------- 2115
H.sapiens N3 KLTLACPGPL ADSSVTLSPV DSLDSPRPFG GP-------- 2114
D.rerio N3 KLTLDMQSAL LDSSVTLSPV DSLDSPR--G GASNAGYVTN 2138
G.aculeatus N3 KLTLDMQSAL LESSVTLSPV DSLDSPH--G GASNAGYITN 2144
Consensus KLTLXXXXXL XDSSVTLSPV DSLDSPRPFG GXSNAGY-TN

Supplementary Figure 4: Notch canonical signalling is not required for Notch3-induced cell death. a, Western blot analysis of Notch1, Notch3 and Jag-1 expression of HUVEC and HUAEC cells and of Notch3 in HUVEC cells treated with DAPT (4μM). **b**, HUVEC cells were electroporated with a GFP expressing plasmid for 24h before being imaged. **c**, Sub-G1 analysis of HUVEC electroporated with a control plasmid (Control), or a plasmid expressing the full-length version of Notch3 or N3ICD for 24h. **d**, HUVEC were electroporated with plasmids expressing a shRNA control (shcontrol) or a shRNA targeting Notch3 (shNotch3) for 24 hours. Cells were then trypsinised and put in Matrigel. YO-PRO®-1 iodide was added 9 hours later to each well 30 min before imaging. Quantification of three independent experiments, mean +/- SD, unpaired t-test). **e**, Scheme representing the different versions of Notch3 used in the figure 3. S1-Cter (aa1573-Cterminus), S2-Cter (1631-Cterminus) and N3ICD (1664-Cterminus) are represented along with Notch3. **f**, HEK293t cells were transfected with the indicated constructs together with a pGL3-Renilla construct and a pGL3-CBF1-Firefly constructs for 48 hours before being assessed for the luciferase expression. **f**, Luciferase activity of HEK293t cells transfected with a pGL3-Renilla construct and a pGL3-CBF-1-Firefly construct together with a plasmid expressing N3ICD or S1-Cter construct and HUVEC electroporated with a pGL3-Renilla construct and a pGL3-CBF1-Firefly construct together with an empty vector, N3ICD, DNMAML, CBF-VP16, S1-CterN3 or an S1-Cter WFP/LAA mutant construct for 48h. **g** and **h,** Western blot performed on HUVEC cells electroporated with the indicated constructs for 24 hours. **i and j**, S1-Cter Notch3 WT (S1-CterN3) or an S1-Cter version of Notch3 in which aspartic acids 2104 and 2107 have been mutated to asparagines (2104/2107) or a S2-Cter Notch3 construct with the same mutation or not were transfected in HEK293t cells treated with a inhibitor of caspase-3 (DEVD-fmk, 5μM), an inhibitor of caspase-9 (LEHD-fmk, 5μM), an inhibitor of caspase-8 (IETD-fmk, 5μM) or a pan-caspase inhibitor (Z-VAD-fmk, 5μM) and analysed by Western Blot for HA (C terminal tag). **k,** HEK293T cells were transfected with a plasmid expressing the intracellular domain of Notch3 (N3ICD) or a N3ICD version mutated for aspartic acids 2104 and 2107 (N3ICD 04/07) together with a pGL3-Renilla construct and a pGL3-CBF1-Firefly construct for 48 hours before being assessed for the luciferase activity. **l**, Alignment of the Notch3 receptor from mouse (M.musculus), Human (H.sapiens), zebrafish (D. rerio) and stickleback (G. aculeatus).

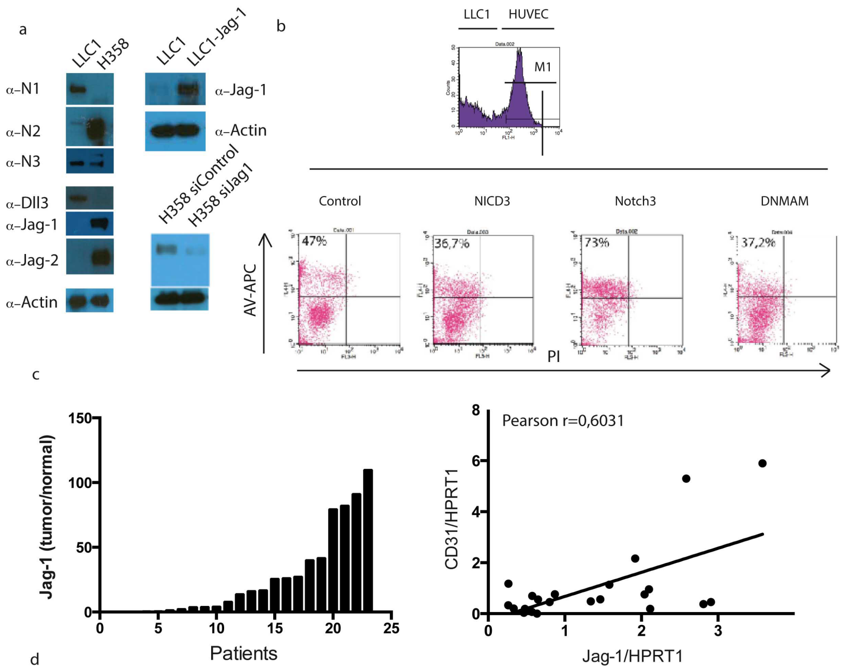

d

	Heyl	hey1	hes1
GSE7670	-0.32819462	0.47323975	-0.37501145
GSE10245	0.01023288	0,49511392	0,04959597

e

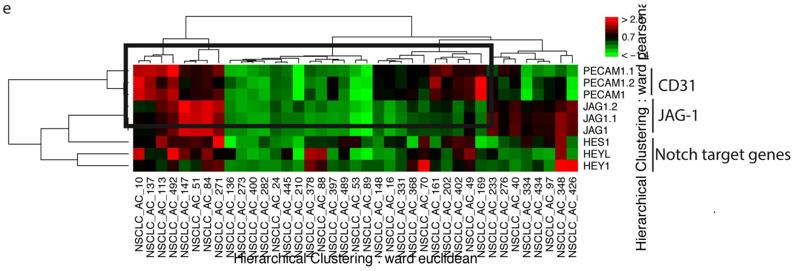

f

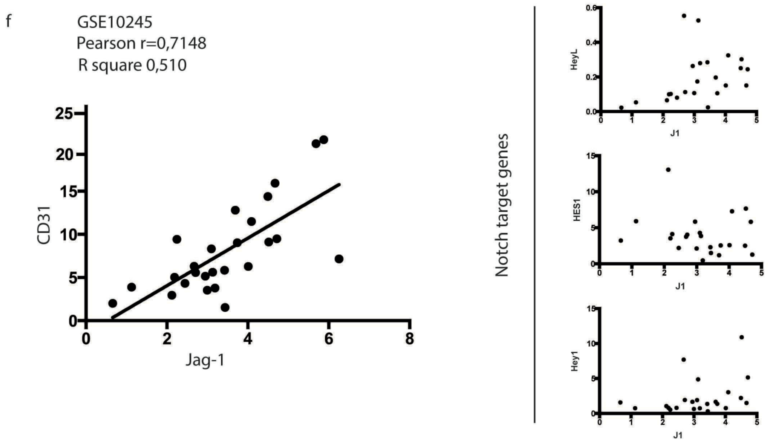

Supplementary Figure 5: Jag-1 rescues Notch3-induced endothelial cells. a, Western blot analysis of LLC1 and H358 cells for Notch1, Notch2, Notch3, Dll3, Jag-1 and Jag-2 expression and of Jag-1 expression in LLC1 transfected or not with Jag-1 expressing construct or H358 transfected with siRNA control or with a siRNA targeting Jag-1. **b,** Co-culture of CMFDA cellTracker green stained HUVEC electroporated with the indicated plasmids and LLC1 were stained with Annexin V-APC and studied by flow cytometry. HUVEC were gated (M1) according to FL1 staining and Annexin V (FL4) positive cells were quantified among HUVEC. Number of Annexin V positive HUVEC cells is specified in each condition. **c**, Tumour/normal tissue ratio of 23 Clear Cell Renal Cell Carcinoma of Jag-1 expression and correlation of PECAM-1 (CD31) mRNA expression with Jag-1 mRNA expression. **d,** Pearson correlation coefficient between Jag-1 and Notch target genes Heyl, Hey1 and Hes1 in non small cell lung adenocarcinoma from the GSE7670 and GSE10245 datasets. **e,** Expression pattern of the three CD31 (PECAM1) probes and the three Jag-1 probes and from Notch target genes HES1, HEY1 and HEYL probes were extracted from the GSE10245 dataset. R package EMA was used to establish the non supervised clustering on gcRMA-calculated Signal intensity provided for each probe. **f,** Correlation between Jag-1 and CD31 or Notch target genes HeyL, Hey1 and Hes1 from patients represented in the black box in **e**.

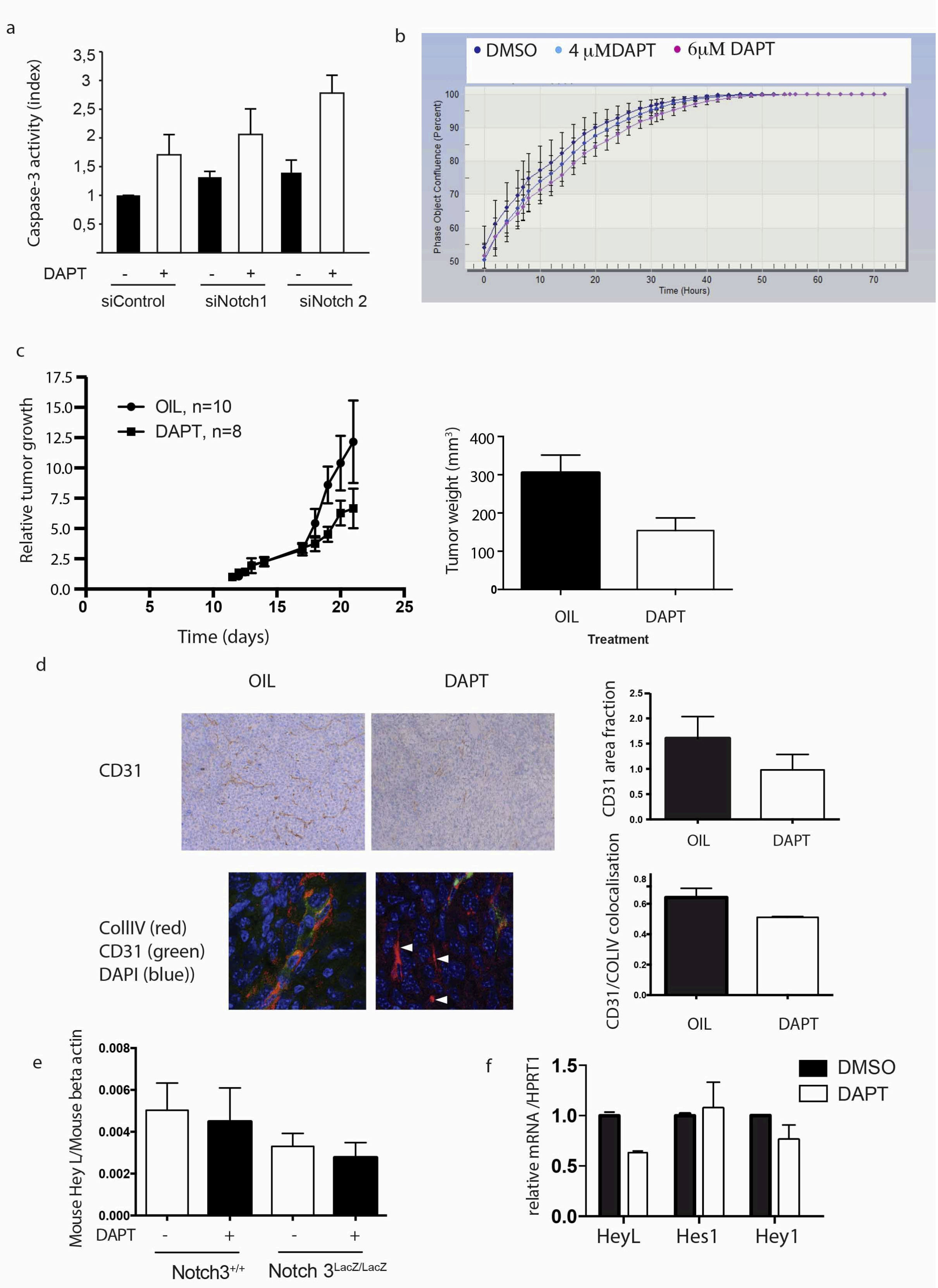
a
Caspase-3 activity (index)
3,5
3
2,5
2
1,5
1
0,5
DAPT
- + - + - +
siControl
siNotch1
siNotch 2
b
DMSO
4 μMDAPT
6μM DAPT
Phase Object Confluence (Percent)
100
90
80
70
60
50
0 10 20 30 40 50 60 70
Time (Hours)
c
Relative tumor growth
17.5
15.0
12.5
10.0
7.5
5.0
2.5
0.0
OIL, n=10
DAPT, n=8
0 5 10 15 20 25
Time (days)
Tumor weight (mm³)
400
300
200
100
0
OIL
DAPT
Treatment
d
OIL
DAPT
CD31
CollIV (red)
CD31 (green)
DAPI (blue))
CD31 area fraction
2.5
2.0
1.5
1.0
0.5
0.0
OIL
DAPT
CD31/COLIV colocalisation
0.8
0.6
0.4
0.2
0.0
OIL
DAPT
e
Mouse Hey L/Mouse beta actin
0.008
0.006
0.004
0.002
0.000
DAPT
- + - +
Notch3+/+
Notch 3LacZ/LacZ
f
relative mRNA /HPRT1
1.5
1.0
0.5
0.0
DMSO
DAPT
HeyL
Hes1
Hey1

Supplementary Figure 6: DAPT treatment induces regression of tumour vascularization. **a**, Caspase-3 activity assay of HUVEC electroporated with control siRNA (siControl), Notch1 siRNA (siNotch1) or Notch2 siRNA (siNotch2) for 48 hours and treated with DAPT (4μM). **b**, Growth curve of LLC1 cells treated with DMSO (1/1000) or 4 or 6 μM DAPT was measured every two hours with the Incucyte Zoom. **c**, $5x10^5$ LLC1 cells were implanted into the left flank of Wild Type C57Bl/6 mice and injected intraperitoneally with 10μl/g of ethanol-corn oil (1/9) or 10μl/g of 1mg/ml DAPT (n=8) on days 11,12,13,14 and 17,18,19,20. Mice were sacrificed on day 21. **d,** Immunohistochemistry for CD31 expression or immunofluorescence for CD31/collagen IV costaining was performed on tumour sections from wild-type mice treated (DAPT,n=8) or not (OIL,n=10) with 10mg/kg of DAPT. CD31 expression and CD31/Collagen IV co-localization was quantified using imageJ. **e,** mRNA was extracted from tumours dissected after 21 days of growth from wild-type C57Bl/6 mice ($N3^{+/+}$, n=6) or Notch3 mutant mice $N3^{LacZ/LacZ}$ C57Bl/6 littermates ($N3^{LacZ/LacZ}$, n=5) treated or not with DAPT. Quantitative RT-PCR was performed to measure HeyL mRNA expression (means +/- SD). **f**, mRNA was extracted from purified tumour-associated endothelial cells from nodules dissected from $Kras^{+/G12D}$ mice treated for 24h (DAPT) or not (DMSO); (n=2 tumours).
